# Factors Affecting the Survival of Ram Spermatozoa during Liquid Storage and Options for Improvement

**DOI:** 10.3390/ani12030244

**Published:** 2022-01-20

**Authors:** Natalie Rizkallah, Caitlin G. Chambers, Simon P. de Graaf, Jessica P. Rickard

**Affiliations:** School of Life and Environmental Sciences, Faculty of Science, University of Sydney, Sydney, NSW 2006, Australia; caitlin.chambers@sydney.edu.au (C.G.C.); simon.degraaf@sydney.edu.au (S.P.d.G.); jessica.rickard@sydney.edu.au (J.P.R.)

**Keywords:** semen preservation, sheep, room temperature, viability, egg yolk

## Abstract

**Simple Summary:**

The success of semen preservation is vital for the use of artificial reproductive technologies in sheep. However, reduced temperatures can cause significant damage to the sperm cell. Recent investigations in other species have identified room-temperature liquid storage as a viable alternative if spermatozoa are protected from the increased risk of lipid peroxidation, a side effect of unaltered metabolism. The following review aims to summarise the factors which contribute to the survival of ram spermatozoa during liquid storage and the role of pro-survival factors and antioxidants in helping to ameliorate the damaging effects caused by lipid peroxidation on fertility. This would contribute towards establishing a new method of semen preservation for the sheep industry which maximises fertility following storage and artificial insemination.

**Abstract:**

Semen preservation is an essential component of reproductive technologies, as it promotes genetic gain and long-distance semen transport and multiplies the number of ewes able to be inseminated per single ejaculate. However, the reduced temperature during cold storage at 5 or 15 °C inflicts sub-lethal damage to spermatozoa, compromising sperm quality and the success of artificial breeding. New and emerging research in various species has reported the advantages of storing spermatozoa at higher temperatures, such as 23 °C; however, this topic has not been thoroughly investigated for ram spermatozoa. Despite the success of storing spermatozoa at 23 °C, sperm quality can be compromised by the damaging effects of lipid peroxidation, more commonly when metabolism is left unaltered during 23 °C storage. Additionally, given the biosafety concern surrounding the international transport of egg-yolk-containing extenders, further investigation is critical to assess the preservation ability of synthetic extenders and whether pro-survival factors could be supplemented to maximise sperm survival during storage at 23 °C.

## 1. Introduction

Semen preservation is defined as the lengthening of the fertile lifespan of spermatozoa by maintaining the functional, ultrastructural, and biochemical properties of the spermatozoa [1]. By prolonging the fertile lifespan of spermatozoa, ejaculate efficiency per elite ram improves, allowing semen to be transported across greater distances and to inseminate more ewes [2], ultimately accelerating genetic gain [1,3].

Semen preservation involves storing spermatozoa in either a liquid or cryopreserved state [4]. Liquid preservation of spermatozoa involves slowing biochemical function by cooling spermatozoa to temperatures between 0–15 °C. However, the fertility of chilled or fresh ram spermatozoa post liquid preservation is limited to 24 h, with an average decline of 10–35% in fertility per day using cervical insemination [1]. This limit restricts the maximum distance between the sire location and place of insemination [1]. Therefore, producers who wish to take advantage of semen collected from international sires must rely on the frozen storage of spermatozoa. Indefinite storage of semen involves cryopreservation, which completely halts biochemical functioning [5]. Cryopreservation involves freezing semen in straws or pellets at −196 °C using liquid nitrogen [1,6].

Despite extensive development on the type of extenders used [1,7], modification of the cooling and freezing processes [5], and the concentration of spermatozoa which is stored [1], the process can still inflict considerable ultrastructural [6,8,9], biochemical [8,9], and thermal damage [1,6,9], leading to reduced or varied fertility rates post thaw for the industry.

Thermal damage, also known as cold shock, is heightened in rams due to their low intramembrane cholesterol-to-phospholipid ratio present on the sperm membrane [7]. Cold shock damage can reduce ram sperm motility and viability up to 60% post thaw [7]. Furthermore, frozen–thawed ram spermatozoa also struggle to penetrate the ovine cervix and achieve fertilisation, recording a pregnancy rate of only 20–30% following cervical artificial insemination [7,9]. As such, producers must rely on laparoscopic artificial insemination to take advantage of the benefits associated with frozen–thawed spermatozoa, which requires the injection of spermatozoa directly into the uterus (60–80% fertility success) [10,11,12]. This process is expensive, requires specialised skills, and raises significant animal welfare concerns in some jurisdictions. Therefore, research into developing an alternative storage method that preserves ram sperm fertility greater than 24 h and reduces sub-lethal thermal damage would be helpful for the sheep artificial reproductive technology toolbox.

There has been growing interest in using ambient-temperature storage (23 °C) to maintain sperm survival during preservation (Table 1). In particular, studies such as Wusiman et al. (2012) demonstrated that ram spermatozoa stored at 23 °C for 24 h did not differ in viability, acrosome integrity, or mitochondrial membrane potential when compared to spermatozoa stored at 4 °C for 48 h, also achieving a similar pregnancy rate to that of fresh spermatozoa. While the concept of storing ram spermatozoa at 23 °C has been investigated, the study mentioned above by Wusiman et al. (2012) did not report any accompanying sperm motility or kinematic results and only assessed the ability of egg-yolk-based extenders to support sperm function [12]. A systemic review or comparison of the traditional ram sperm extenders and their performance at different temperatures would be beneficial in validating this storage technique for the industry (Table 1). Furthermore, despite these studies reporting encouraging results on the impact of 23 °C storage on spermatozoa, many studies also highlighted an accentuated risk of lipid peroxidation damage when stored at 23 °C [13,14].

Lipid peroxidation is a metabolic process where reactive oxygen species (ROS) are formed by the oxidative degeneration of polyunsaturated fatty acids [13]. This process is accelerated at higher temperatures (as metabolism occurs unrestricted, leading to ROS overproduction [4,14]) and negatively impacts sperm function [14,15].

Reduced lipid peroxidation damage has been reported in some species such as stallions [16], rams [17,18], and buffalo [19] when extenders are supplemented with pro-survival factors, e.g., L-carnitine, pyruvate, and/or melatonin. However, research fully defining the antioxidant-like effect of these pro-survival factors in ram spermatozoa during 23 °C storage is limited. Therefore, research into these factors’ ability to minimise lipid peroxidation and reduce ROS while supporting sperm functionality at 23 °C is required.

As such, this literature aims to review the current knowledge on the factors that impact the quality of ram spermatozoa during liquid preservation at 5, 15, and 23 °C, with a focus on temperature-induced liquid peroxidation at room temperature (23 °C). Additionally, methods to reduce the deleterious effects of lipid peroxidation on ram spermatozoa following storage at 23 °C will be explored, including the supplementation of the pro-survival factors L-carnitine, pyruvate, and melatonin. The findings examined in this review will help identify the components required to preserve the quality and lifespan of ram spermatozoa following liquid storage at 23 °C, offering the ovine industry an alternative to the cryopreservation of spermatozoa.

## 2. Factors Affecting the Survival of Spermatozoa during Liquid Storage

During semen preservation, various factors such as temperature, storage concentration, and extender can influence sperm quality and survivability during storage. When developing a new semen preservation technique or extender, understanding the relationship between storage conditions and sperm quality is crucial to prevent sperm damage. Therefore, an in-depth examination of temperature, the concentration of sperm, and the extender used on the survivability of sperm during storage is presented below.

### 2.1. Temperature

**Figure 1:**
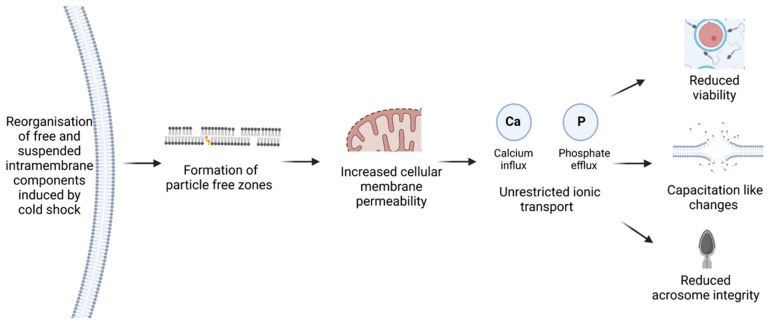
Summary of cascading membrane damage induced by cold shock in ram spermatozoa stored at temperatures below 18 °C. Adapted from [4,11]. Created on Biorender.com.

Cold shock or thermal damage of sperm occurs when the ambient temperature surrounding the sperm cell is reduced rapidly, significantly influencing the organisation of both suspended and free intramembrane components [27]. Cold shock damage is a significant consequence of the reorganisation of these intramembrane components, which creates lipid–lipid agglutinations, disrupts cell signalling, and forms particle-free zones [27,28]. These particle-free zones reduce membrane integrity and selective permeability, allowing unrestricted ionic transport across the membrane (Figure 1); [28]. this impairment has been reported in boar spermatozoa, recording a viability of 36.7% and an acrosome integrity of 20.2% following storage at 5 °C for 4 h. These results are significantly lower than that of freshly stored boar spermatozoa, which demonstrated 74.4% viability and a 44.2% acrosome-intact population [29]. Studies such as these demonstrate the significant impact reduced temperatures have on the quality of preserved spermatozoa and the need for optimising these methods.

Ram spermatozoa are highly susceptible to temperature changes, as they contain a low intramembrane cholesterol-to-phospholipid ratio compared to other species. In contrast to this, as seen in Table 2, boar spermatozoa can acquire partial resistance to cold shock during storage even though they have a low cholesterol-to-phospholipid ratio [30,31]. Bull spermatozoa are highly resistant to cold shock, as they contain the highest cholesterol-to-phospholipid ratio of 0.45 [30]. The correlation between cold shock damage and cholesterol-to-phospholipid ratio is further demonstrated within species, as boar spermatozoa with low and high membrane fluidity were correlated, respectively, with 38.8 ± 2.5% and 26.8 ± 3.2% cholesterol levels [32]. The extra intramembrane cholesterol protects against cold shock by supporting membrane stability [33]. It does this by limiting particle movement within the membrane during the cooling process, reducing membrane destabilisation events by 30% [32]. As ram spermatozoa have low cholesterol content and are therefore highly susceptible to cold shock damage, developing a semen preservation technique that aims to hold spermatozoa above cold-shock-inducing temperatures and provide extra membrane stability could preserve sperm quality for longer periods and make liquid storage at higher temperatures a more viable option for farmers.

### 2.2. Concentration of Spermatozoa

The recommended standard concentration for liquid ram spermatozoa storage at 23 °C is not fully defined as an international set standard, unlike frozen or chilled liquid storage (5–15 °C) [7,38]. However, there have been many studies demonstrating the effects of high dilutions on spermatozoa, referred to as the dilution effect. The dilution effect occurs when spermatozoa undergo osmotic stress due to high dilution ratios, hypothesised to be related in part to a reduction in seminal plasma [39,40,41]. Gungdogen et al.’s, 2010 investigation demonstrated these consequences when ram spermatozoa were diluted to 100 × 10^6^ sperm/mL and recorded lower membrane damage and oxidative stress compared to spermatozoa diluted to 25 × 10^6^ sperm/mL. Research has demonstrated this result in other species with various sperm concentrations, including rabbits [42], cattle [43,44], bucks [45], and pigs [46].

Research has identified various methods to reduce the dilution effect during semen preservation, one of which is slowing the dilution rate by using a step-wise dilution process and selecting suitable extenders. Step-wise dilutions allow spermatozoa time to osmotically balance with the changing external environment, preventing sudden osmotic stress [7,41]. By optimising extender ingredients and osmolarity, the spermatozoa’s external environment is minimally altered [10]. Despite this knowledge, the exact mechanisms of the dilution effect in ram spermatozoa have not been fully elucidated, highlighting a knowledge gap within the semen preservation industry.

### 2.3. Extenders

Extenders are responsible for protecting and providing adequate resources to maintain sperm survival throughout the various stages of semen preservation. Extenders protect spermatozoa in many ways, including stabilising the plasma membrane and maintaining intracellular and intramembrane ionic concentrations, thus reducing cold shock damage and osmotic shock [1,47,48,49,50]. Extenders comprise diverse vital ingredients, allowing them to cater to species-specific requirements and the preservation method utilised.

Sperm cells are biologically different between species and thus require specific ingredients for protection. Furthermore, each species has a unique sperm pH tolerance zone where peak respiration is achieved [5,41]. For rams, this pH zone falls between 7.3 and 7.5, whereas bulls and cocks fall, respectively, between 6.5–7 and 6.9–7 [51]. These biological differences are not the only factor contributing to the different makeup of extenders, as each storage technique has particular stressors which sperm extenders must consider.

As such, extenders are specific to storage type depending on the key ingredients they contain. For example, Salamon’s egg-yolk-based cryoprotectant contains glycerol to protect spermatozoa against ice crystal formation during the freezing process and uses glucose as a sugar source; however, Salamon’s egg-yolk-based chilled extender does not contain glycerol and uses fructose [5], stimulating metabolism through a different pathway. Therefore, before storage at 23 °C can be optimised, the current ram extenders and their ingredients should be examined for their ability to support sperm function at 23 °C. Research must consider the major factors or features spermatozoa will require when held at higher temperatures to maximise fertility following storage.

#### Key Extender Ingredients

Standard in-house ram liquid extenders used to protect spermatozoa from changing temperatures, cold shock, and osmotic shock include Tris–citrate–fructose (TRIS), Salamon’s egg-yolk-based extender (EY), and phosphate-buffered saline (PBS) [10]. Some key ingredients include phosphate-buffered saline, tris(hydroxymethyl)aminomethane (Tris), fructose, citric acid, egg yolk, bovine serum albumin (BSA), and various antibiotics. For example, BSA and Tris aid spermatozoa by acting as a protein source and a pH buffer [1], whereas fructose supplies sugars that provide crucial substrates for metabolism [26,52,53,54]. Additionally, citric acid acts as a pH buffer, maintaining peak respiration metabolic rates [5,10], and egg yolk provides membrane support by supplying low-density lipoproteins (LDL) and cholesterol [33,52]. Finally, antibiotics including penicillin, streptomycin, and gentamycin aid spermatozoa by controlling bacterial growth [53]. Together, all these ingredients create liquid ram sperm extenders that can minimise the risk of any storage-related damages that compromise sperm quality.

Currently, it is suggested that the primary storage-related damages for 23 °C include oxidative stress and bacterial growth [12]. Therefore, the development of a ram-specific extender for 23 °C needs to include key ingredients that not only preserve sperm quality but also provide extra support to reduce oxidative damage and bacterial growth.

## 3. Changes to Spermatozoa during Liquid Preservation

**Figure 2:**
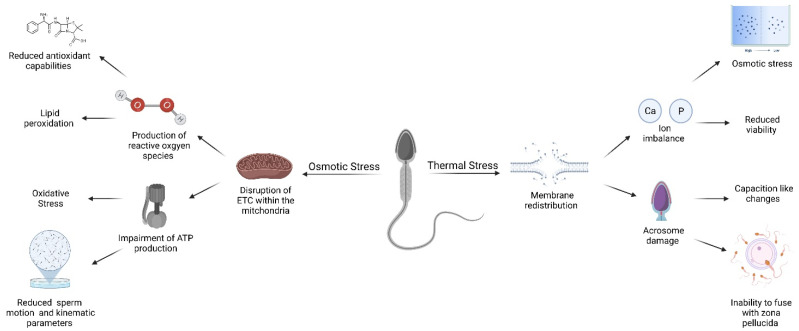
Summary of significant structural, functional, and molecular impairments incurred by ram spermatozoa cells undergoing osmotic and thermal stress inflicted by storing indefinitely via cryopreservation or temporarily chilled to 5–15 °C. Adapted from [1,4] and created on BioRender.com.

Despite ensuring the appropriate storage conditions during storage, it is common for spermatozoa to still incur some degree of osmotic, biochemical, and thermal stress [55]. These stressors force the cell to adapt and undergo a series of delicate conformational and metabolic changes. Most of these stressors cause membrane redistribution, lipid peroxidation, and impaired ATP production and motility (Figure 2 and Table 3). The following section will discuss these stressors, focusing on the resultant lipid peroxidation and its common occurrence following liquid storage at room temperature (23 °C).

### 3.1. Membrane Redistribution

A natural thermotropic transition involves the redistribution of intramembranous components to adapt to the surrounding environment [56]. This transition occurs during maturation or as a defence against reducing temperatures [3,8]. Throughout preservation, spermatozoa go from 37 °C post ejaculation to 23 °C for dilution and then to either 15 °C, 5 °C, or −196 °C for final storage [22]. Incorrect cooling procedures induce early thermotropic phases, leading to significant membrane damage. As seen in Table 3, this damage includes a wide variety of consequences; however, it primarily includes membrane damage, such as protein reconfiguration and lipid agglutination, which creates irreversible particle-free zones known as ultrastructure freeze fractures [8].

Holt and North’s 1986 study was one of the first to document thermotropic phases in rams. Ram spermatozoa displayed midpiece lipid agglutinations and acrosomal particle-free zones due to temperature-induced thermotropic phases [27]. This reconfiguration led to many ultrastructural and biochemical changes, presented in Table 3. These damages are similar in boars; however, due to higher cholesterol concentration, the intramembrane lipid agglutinations were localised around the head rather than the neck, as seen in rams [27]. Furthermore, bull spermatozoa demonstrated minimal particle-free zones or membrane redistribution [57]. These differences reflect the varying protecting nature of intramembrane cholesterol concentrations against cold shock, as rams, who have low intramembrane cholesterol, demonstrated more severe particle redistributions than bulls, which have a higher intramembrane cholesterol concentration. Particle-free zones are harmful to spermatozoa, as they contribute to membrane leakiness, encouraging an imbalance of homeostatic ions particularly heightened in rams (Table 3).

A direct consequence of particle-free zones is the disruption of homeostatic ion concentrations, leading to membrane collapse [58]. Disrupted ion balance is detrimental, as ions act as cellular signals responsible for initiating capacitation-like changes, disrupting metabolic pathways, and halting the overproduction of cytotoxic by-products [35,59]. As seen in Table 3, a harmful ionic event following cold shock in ram spermatozoa is the influx of calcium and efflux of phosphate at 17 °C [58]. This event both stimulates the enzyme ATPase and reduces available intracellular phosphates, disrupting the mitochondrial electron transport chain, reducing ATP production, and increasing the frequency of escaped electrons, contributing to lipid peroxidation [31,59,60]. By understanding the changes associated with particle-free zones, extenders can be equipped to protect spermatozoa from these damages, primarily when held or maintained at higher temperatures.

The development of extenders and storage conditions have previously used the knowledge of thermotropic phases to accommodate the various stressors to which spermatozoa are subjected during semen preservation. For example, in rams, it was identified that intramembranous redistribution begins at a higher temperature during cooling due to low-cholesterol environments [58]. Therefore, adding cholesterol to an extender could reduce the damage incurred by colder temperatures, preserving motility and sperm quality for longer [27,58]. Furthermore, membrane redistribution can be significantly reduced if spermatozoa are stored above 17 °C [58]. Therefore, research into methods of storing liquid ram spermatozoa at 23 °C could reduce cold shock damages, preserving sperm function and quality for longer than 24 h.

### 3.2. Disruption of Respiration and Subsequent Decrease in Motility

In rams, respiration, which powers motility, occurs via the mitochondrial electron transport chain (METC), fructolysis, or glycolysis [5,60,61,62]. METC yields a higher ATP per mol of glucose when compared to glycolysis, proving to be a more efficient process [63]. However, around 70% of all ATP consumption is used to power motility [64] 

ATP production is impaired when incorrect storage conditions allow the METC to proceed unrestricted, eventually leading to the exhaustion of METC resources and dysfunction of the METC complexes of up to 75% [62,64]. For example, the Bilodeau et al., 2002 study reported that excess hydrogen peroxide produced from incorrect storage and METC dysfunction decreased ATP concentrations from 244 ± 53 pmol/10^6^ cells and 4.83 ± 1.08 µm to 10 ± 4 pmol/10^6^ cells and 2.80 ± 0.99 µm in bovine sperm following storage for 6 h at 38 °C.

Even though higher storage temperatures support motility, they can also exhaust energy resources, shortening overall lifespan. Therefore, by understanding how ATP production and motility are linked at higher temperatures, extenders could be built to maintain the mitochondrial METC while suppressing ROS production, allowing sperm to preserve motility.

### 3.3. Lipid Peroxidation and Production of Reactive Oxygen Species

**Figure 3:**
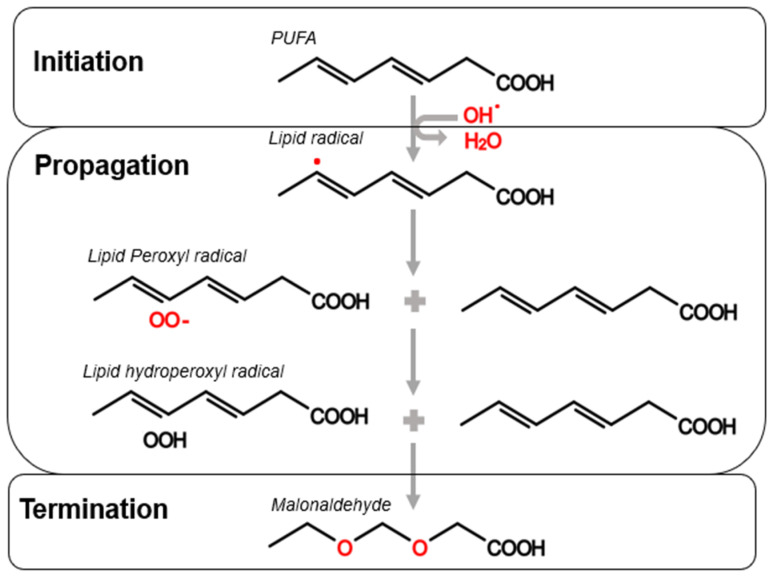
Summary of the three steps involved in lipid peroxidation: initiation, propagation, and termination. Adapted from [13,67,69].

Lipid peroxidation is one of the significant causes of sperm dysfunction following storage at 23 °C. This process creates hydroperoxide intermediates and lipid peroxyl radicals through the oxidative degeneration of lipid metabolism [4,59]. Lipid peroxidation (Figure 3) contains three processes: initiation, propagation, and termination, which together contribute to sperm dysfunction [4,15].

This section briefly summarises the biochemistry of lipid peroxidation and the significant effect of lipid peroxidation on ram sperm motility, viability, and acrosome integrity during storage at 23 °C.

#### 3.3.1. Lipid Peroxidation Biochemistry

The first step of lipid peroxidation, initiation, involves free radicals breaking off allylic hydrogens within polyunsaturated fatty acids and forming carbon-centred lipid radicals [4,65]. As seen in Figure 3, the propagation step begins with the lipid radical combining with diatomic oxygen molecules and creating lipid peroxyl and hydroperoxyl radicals. It also involves the production of more polyunsaturated fatty acids, which goes on to repeat the initiation step, creating an autocatalytic, self-propagating reaction [65,66,67]. These two steps are where lipid peroxidation damage occurs, as initiation breaks down lipid membrane bilayers, and propagation produces lethal ROS, which directly inhibits the METC and oxidative phosphorylation [4]. The length of the initiation and propagation stages depends on the natural antioxidant capabilities of spermatozoa to initiate termination [4].

Antioxidants bring upon the final stage of lipid peroxidation, known as termination (Figure 3). They do this by neutralising radicals via the donation of hydrogen molecules to oxygen molecules and preventing further reactions [4]. As seen in Figure 3, termination can also be activated by radicals self-reacting and forming stable cytotoxic products such as malonaldehyde [68]. Future sections will extensively discuss natural and artificial antioxidants used in semen preservation to combat lipid peroxidation effects during storage at 23 °C.

#### 3.3.2. Impact of Lipid Peroxidation on Sperm Function

Motility and membrane integrity are significantly hindered by lipid peroxidation during 23 °C storage. Motility is hindered because ROS reconfigures METC enzymes, creating inefficiencies throughout the METC and increasing the frequency of electron leakiness [65,66]. An example of this occurring is 4-hydroxy-2-nonenal reacting with the mitochondrial protein succinic acid dehydrogenase [70]. This reaction changes the activation site of succinic acid from the carboxyl to the β carbon, preventing it from undergoing catalyst reactions needed for oxidative phosphorylation [70]. This effect contributes to impaired motility by reducing ATP production and increasing escaped electrons that contribute to lipid peroxidation [66,67].

Lipid radicals favour high-polyunsaturated-lipid environments such as lipid bilayers. Membrane integrity and selective permeability are lost by radicals attacking the structure of membranes, inducing oxidative stress and unrestricted ion transport [69]. This combination impairs many sperm functions such as metabolism, motility, and fertility [71]. To protect themselves against the damaging effects of lipid peroxidation during 23 °C storage, spermatozoa can utilise endogenous and exogenous antioxidants.

#### 3.3.3. Natural Defences to Reduce Lipid-Peroxidation-Induced Damage during Liquid Storage

Spermatozoa are naturally provided with enzyme-based and non-enzyme-based antioxidants within the seminal plasma and harbour small intracellular antioxidants within the cytoplasm [44]. For example, enzyme-based antioxidants include catalase, glutathione S peroxidase (GSH peroxidase), and superoxide dismutase, whereas non-enzyme-based antioxidants can include cysteine vitamin E and methionine [4]. These antioxidants work concomitantly to neutralise radicals and prevent further oxidation reactions [72]. The protective ability of antioxidants relies on their concentration, as when an imbalance of antioxidants and ROS production occurs, lipid peroxidation begins [73].

In ram spermatozoa, glutathione-based antioxidants are higher in concentration than catalase and superoxide dismutase [17,74]. This concentration difference peaks during the breeding season, seen when Casco et al. (2010) recorded superoxide dismutase, catalase, and GSH peroxidase concentrations of 8.86 ± 0.015 nmole/min.mL, 2.11 ± 0.25 μmole/min.mL and 61.57 ± 4.48 nmole/min.mL, respectively, during the breeding season and 8.56 ± 0.26 nmole/min.mL, 1.74 ± 0.19 μmole/min.mL and 52.86 ± 8.53 nmole/min.mL, respectively, during the non-breeding season. Furthermore, Hamilton et al.’s, 2016 study demonstrated a positive correlation between GSH peroxidase concentration and the rate of lipid peroxidation in chilled ram spermatozoa; however, the study found no correlation between superoxide dismutase or catalase and lipid peroxidation [74].

The protective abilities of natural antioxidants are not sufficient for storage at 23 °C due to the limited biosynthetic capacity of spermatozoa and diluted concentration of natural antioxidants [4,75]. Research into exogenous antioxidant supplementation to extenders can close the gap between antioxidants and ROS, protecting sperm from lipid peroxidation at 23 °C and making a viable alternative for storing ram spermatozoa [4].

## 4. The Use of Antioxidant Supplementation to Attenuate Lipid Peroxidation Stress and Promote Liquid Spermatozoa Survival during Storage at 23 °C

Extenders are supplemented with exogenous antioxidants to resupply antioxidant defences against oxidative stress during storage at 23 °C [41]. Investigations into the advantages of supplying exogenous antioxidants to spermatozoa extenders have been successful in preserving sperm quality [76].

The advantages of exogenous antioxidants work in a dose- and temperature-dependent fashion [68,77]. For example, catalase illustrates dose dependency for rams, as 50 U/mL of catalase improved acrosome integrity, whereas 100–200 U/mL of catalase improved total motility, while 200 U/mL of catalase was cytotoxic to spermatozoa [73,78]. Maxwell and Stojanov’s 1996 reports on temperature dependency showed that ram spermatozoa diluted with superoxide dismutase and catalase preserved greater motility at 15 °C when compared to 25 °C. Research into specific exogenous antioxidants for use in the ram to maximise survival during storage at 23 °C has not yet been fully elucidated and highlights a significant knowledge gap within the semen preservation industry.

Research into the pro-survival factors L-carnitine, pyruvate, and melatonin has shown promise in protecting spermatozoa at a wide variety of storage temperatures, including 23 °C.

### 4.1. L-Carnitine

L-carnitine (LC) is a quaternary ammonium compound that acetylates into acetyl-L-carnitine (ALC) by the actions of carnitine acyltransferase 1 [79,80,81,82,83]. LC’s primary responsibility is to facilitate the transport of exogenous fatty acyl-CoA across the mitochondrial matrix [80]. In comparison, ALC acts as a readily oxidizable energy source for respiration as well as a buffer for acetyl-CoA transportation [79,80,81,82,83]. Both molecules are vital for the maintenance of metabolic processes such as motility.

Previous research has demonstrated that these roles improve the motility of sperm stored at higher temperatures in species such as boars [84], bucks [85], stallions [55], bovines [86], and rams [87] (Table 4). LC protects sperm motility mainly by sustaining METC, ATP production, and membrane integrity while providing antioxidant defences during storage [58]. For example, after ten days of storage, 50 mM of LC added to boar spermatozoa stored at 17 °C had increased motility, mitochondrial activity, and ATP production, as well as antioxidant capacity [84]. Despite this success, some studies, such as Deana et al. (1989) (Table 4), demonstrated that the addition of 20 mM LC can be harmful to bovine sperm spermatozoa, while the addition of 20 mM of LC or ALC at either 5 °C, 20 °C, or 37 °C storage maintained 80% viability but increased intracellular calcium by 24%, which decreased progressive motility and oxygen consumption after 24 min (Table 4) [86]. These results were strongly correlated with higher temperatures, as extracellular LC or ALC uptake was demonstrated to be higher at 37 °C and 20 °C when compared to 5 °C [86]. There is reason to investigate LC’s other beneficial or inhibitory effects on ram spermatozoa, particularly its antioxidant ability at 23 °C.

LC and ALC act as antioxidants by forming a part of a negative feedback loop with the METC, which reduces radical synthesis and lipid peroxidation [83]. Recent investigations into LC support this, as the addition of 7.5 mM LC reduced ROS production by 40–60% in liquid stallion spermatozoa following storage at 23 °C [55]. Furthermore, presented in Table 4, 7.5 mM of LC maintained membrane intactness in ram spermatozoa stored at 15 °C for 96 h [88]. Further details of studies which have investigated the use of LC in preserving sperm functionality during liquid storage are presented in Table 4. These studies indicate that 10 mM of LC could potentially be the optimal supplementation concentration; however, future research is needed to confirm this. Although previously researched for cryopreserved ram spermatozoa [89], the effect of LC and ALC as an antioxidant protecting ram spermatozoa against lipid peroxidation when stored at 23 °C has not been thoroughly investigated, highlighting a significant knowledge gap in higher-temperature semen preservation. 

### 4.2. Pyruvate

Pyruvate sustains the quality of liquid-stored spermatozoa by maintaining the efficiency of the metabolic pathways [63]. Pyruvate supplies metabolic pathways by transforming into the metabolic intermediates acetyl-CoA, malate, and oxaloacetate [90,91]. The functioning of these processes is crucial to maintaining motility during liquid storage [90]. Studies have demonstrated this by utilising antimycin A to inhibit human spermatozoa’s metabolic functioning and motility [61]. With the addition of pyruvate to these inhibited pathways, ATP production and progressive motility increased by 56% and 21%, respectively [91]. In addition, stallion spermatozoa stored at 23 °C for 72 h demonstrated that 10 mM of pyruvate increased the total motility of spermatozoa from 18.9 ± 1.4% in control to 26.1 ± 1.7% [55]. Apart from increasing motility, pyruvate also reduces electron leakage and prevents the onset of lipid peroxidation, thereby giving it antioxidant qualities [92]. For example, as seen in Table 5, Ortiz-Rodríguez et al. (2021) supplemented stallion spermatozoa with pyruvate and stored it at 18 °C for 48 h. They demonstrated an increase in mitochondrial activity from 24.1 ± 1.8% in the control to 51.1 ± 0.7% in high-pyruvate media while maintaining 76.2 ± 1% viability, reducing ROS from 64.3 ± 1.3% to 45.4 ± 4.7%, and increasing GSH concentration from 10,162 ± 731.7 to 15,553 ± 912.

Pyruvate reduces the onset of lipid peroxidation and ROS production during liquid storage by behaving as both a direct and indirect antioxidant [93]. Pyruvate can directly interact as an antioxidant due to the reactive keto-enol within its conformation [94]. This architecture allows pyruvate to directly neutralise peroxides and peroxynitrites in a non-enzymatical redox reaction without producing cytotoxic oxygen radicals [95]. Furthermore, pyruvate can indirectly stimulate the glutathione pathway through glutathione reductase production [63]. Furthermore, in Table 5, Gibb et al.’s, 2015 study demonstrated that stallion spermatozoa stored at 25 °C and supplemented with 10 mM of pyruvate had reduced lipid peroxidation levels after 72 h.

The benefit of pyruvate during chilled sperm storage has been well-documented (Table 5); however, limited research into its use during higher-temperature storage shows pyruvate’s ability to reduce the onset of lipid peroxidation and ROS production in rams at 10 mM. Therefore, further research into pyruvate’s protective ability at 23 °C is crucial for developing liquid ram sperm-specific extenders for the industry.

### 4.3. Melatonin (N-Acetyl-5-Methoxytryptamine)

Melatonin is acclaimed for its role in circadian rhythm; however, it is growing in popularity as a mitochondrial-targeted antioxidant due to its scavenging abilities and indirect role in stimulating antioxidant gene expression [17]. Melatonin’s success in preventing lipid peroxidation is attributed to its antioxidant radical scavenging abilities [97]. Melatonin’s scavenging abilities are unique, as they differ depending on the dosage supplemented to spermatozoa and the type of ROS it interacts with (Table 6) [98,99,100]. For example, melatonin neutralises hydroxyl radicals (OH) by transforming them into stable metabolites [101]. A reduced lipid peroxidation level was demonstrated in boars when melatonin reduced malondialdehyde (MDA) levels from 17.5 nmol/1 × 10^6^ to 12 nmol/1 × 10^6^ [98]. Similar trends were reported in bulls, as 7 mM of MDA was produced in melatonin-supplemented treatments compared with 11.7 mM MDA produced in the control [102]. Unlike hydroxyl, melatonin does not undergo a direct redox reaction with nitric oxide [91]. Here, melatonin binds with Ca^2+^ and calmodulin, nitric oxide production components, via the eNOS-dependant nitric oxide production pathway [103]. Previous research in rams has demonstrated the benefits of melatonin’s unique scavenging abilities [104]. For example, Casco et al.’s, 2010 investigation concluded that ram spermatozoa supplied with melatonin stored at 39 °C for 3 h demonstrated decreased capacitation and apoptotic-like changes. Previous research into the effects of melatonin during storage in different species is extensively summarised in Table 6, including rams [20], boars [105], and buffalo [19]. Furthermore, new research has also demonstrated that melatonin plays a role in preserving fertility, as natural melatonin concentration ranges from 137.51 ± 17.8 pg/mL in the breeding season to 46.57 ± 8.37 throughout the non-breeding season [17,106].

Previous investigations into melatonin for semen preservation of various species at 23 °C have shown considerable promise in preserving sperm quality for longer than 24 h; however, research is limited for rams, especially at 10 mM. Therefore, investigations into the protective ability of melatonin during 23 °C storage are necessary.

## 5. Conclusions

Semen preservation is a vital artificial reproductive technology, enabling producers to take advantage of the benefits from genetically superior sires. Despite significant developments in preservation methods and extenders, as a result of being subjected to reduced temperatures, the process can still inflict sub-lethal damage to spermatozoa, including integral membrane protein agglutination, altered protein functions, and loss of selective membrane permeability.

The storage of spermatozoa at ambient temperature removes the risk of thermal damage caused by cold shock. It has shown success in maintaining the motility and viability of spermatozoa from a variety of species, including stallions [107], humans [108], mice [109], and bulls [110]. Several studies have investigated the ability of ram spermatozoa to survive storage at 23 °C; however, it has yet to be comparable to current industry methods, and the increased threat of lipid peroxidation and ROS production could significantly hinder fertility post storage.

Ongoing research has shown promising results using exogenous antioxidants such as L-carnitine, melatonin, and pyruvate to combat damage caused by lipid peroxidation. However, limited research has investigated the ability of these antioxidants to protect ram spermatozoa when stored at 23 °C. It would therefore be prudent to not only assess the function of ram spermatozoa following storage at 23 °C when supplemented with the above-mentioned pro-survival factors, but also compare the rates of lipid peroxidation to those produced following preservation at 5 °C and cryopreservation. If liquid storage at 23 °C is to be successful and widely adopted amongst the industry, it needs to reduce the rates of lipid peroxidation compared to other methods of semen preservation and record higher levels of fertility, specifically for artificial insemination.

If ram sperm functionality and fertility could be maintained following storage longer than 24 h at 23 °C, this would provide an alternative sperm storage option for producers when they want to take advantage of genetics from superior sires located vast distances from the insemination site. It would also offer producers an alternative to freezing spermatozoa, reducing the risk of sub-lethal damage and enabling the use of non-surgical artificial insemination methods such as cervical artificial insemination.

## Figures and Tables

**Table 1:** Summary of publications investigating semen storage temperatures between 20–37 °C in various species.

Species	Temp	Extender	Result	Reference
Ram	4 °C 23 °C −196 °C	Citrate–glucose–EY ^1^	Higher fertility at 23 °C and 4 °C when compared to −196 °C at 24 h	[12]
	5 °C 20 °C	Milk-based Sodium-citrate-based Tris–citrate–fructose + EY ^1^	Sodium-based and TRIS with and without EY maintained the highest viability at 72 h	[20]
	5 °C 24 °C	Tes–Tris–fructose solution with EY *	Higher sperm abnormality in sperm stored at 24 °C when compared to 5 °C at 48 h	[21]
			Higher embryo cleavage at 24 °C when compared to 5 °C at 48 h	
	4 °C 20 °C 37 °C	Tyrode’s albumin lactate pyruvate + EY ^1^	Higher TM at 20 °C and 37 °C when compared to 4 °C at 72 h	[22]
Bull	5 °C 18 °C	Cap ^1^ BioXcell INRA96	Higher TM in Cap extender when compared to BioXcell and INRA96 at 72 h	[23]
Buck	5 °C 15 °C 25 °C	PBS supplemented with 10 mM pyruvate or lactase	Higher PM at 15 °C when compared to 5 °C and 25 °C at 168 h	[24]
			Higher viability at 15 °C when compared to 5 °C and 25 °C at 168 h	
Stallion	15–20 °C	INRA96	Lower TM at 20 °C when compared to 5 °C at 12 h	[25]
	5 °C 15 °C 20 °C	SM ^1^ Cap ^1^ NFMS ^1^	Higher fertility PM in Cap than SM and NFM at all temperatures at 72 h	[26]

^1^ EY = egg-yolk-based extender, SM = skim-milk-based extender, Cap = Caprogen, and NFMS = non-fat milk solid extender. * Epididymal sperm.

**Table 2:** Summary of publications investigating semen storage temperatures between 20 and 37 °C in various species.

Species	Cholesterol (mol %)	Ratio of Cholesterol: Phospholipid ^1^	Resistance to Cold Shock	Reference
Ram	27	0.43	Low	[34]
Bull	31	0.45	High	[35]
Stallion	Not reported	0.36	Low	[36]
Boar	Not reported	0.37	Partial	[37]

^1^ Ratio of cholesterol to phospholipids from isolated plasma membrane fractions of spermatozoa.

**Table 3:** Significant thermotropic phases and associated metabolic and physiological effects in the ram and boar spermatozoa.

Phase Transition Temp	Species	Metabolic Changes	Physiological Changes
30–36 °C *	Ram	Intramembrane lipid scattering	Membrane fusogenicity
		Phosphate discontinuity	Increased protein immobility and reconfiguration
23–26 °C	Ram	Calcium transport discontinuity	ATPase activity reduced
			Intramembrane lipid scattering and agglutination
23 °C and 14 °C	Boar	Reduction in -CH_2_ absorbance	Inhibition of partial acquisition of cold shock resistance
18 °C	Boar	40% increase in potassium efflux	Acceleration of ATPase
17 °C	Ram	Intramembrane lipid scattering	Membrane particle redistribution/aggregation Ultrastructural freeze fractures
		Calcium influx	Capacitation initiated Irreversible membrane leakiness

* Adapted from [8,31,37].

**Table 4:** Summary of noteworthy results on the supplementation of L-carnitine (LC) to spermatozoa during storage above 5 °C in various species.

Species	Temp (°C)	Extender	Result	Reference
Boar	17	Androhep	Higher membrane integrity at 50 mM when compared to 12.5 mM, 25 mM, and 100 mM at ten days	[84]
Ram	5	Skimmed milk extender	Higher TM at 10 mM when compared to 1 mM, 2.5 mM, 5 mM, and 7.5 mM at 96 h	[88]
Stallions	23	MBWW	Higher MMP (%) at 0 mM than at 10 mM at 72 h	[55]
			Higher PM at 0 mM when compared to 10 mM at 72 h	
			Higher lipid peroxidation at 0 mM when compared to 10 mM at 72 h	
			Higher ROS at 0 mM when compared to 10 mM at 72 h	
Bovine	5 20 37	Sodium chlorine extender	Lower PM at 20 mM LC and 20 mM ALC when compared to 0 mM and 20 mM NaCl at 72 h	[86]

MMP = mitochondrial membrane potential and MBWW = modified Biggers, Whitten, and Whittingham.

**Table 5:** Summary of noteworthy results on pyruvate supplementation to semen storage above 5 °C in various species.

Species	Temp (°C)	Extender	Result	Reference
Bovine	38.5	TALP ^1^	Higher TM when compared to 1 mM, 2 mM, and 5 mM at 6 h	[63]
			Higher intracellular ATP at 5 mM than at 1 mM at 6 h	
			Higher extracellular ATP at 1 mM than 5 mM at 6 h	
Stallion	18	Tyrode’s	Higher TM at 1 mM when compared to 0 mM at 48 h	[92]
			Higher MMP ^1^ at 1 mM when compared to 0 mM at 48 h	
			Higher ROS at 1 mM when compared to 0 mM at 48 h	
	37	MBWW ^1^	Higher TM at 5.5 mM when compared to 0 mM at 1 h Higher ROS at 5.5 mM when compared to 0 mM at 1 h	[96]
	23	MBWW ^1^	Highest TM at 10 mM when compared to 1.25 mM, 2.5 mM, 5 mM, and 20 mM at 72 h Highest PM at 10 mM when compared to 1.25 mM, 2.5 mM, 5 mM, and 20 mM at 72 h	[55]

^1^ TALP = Tyrode’s albumin lactate pyruvate, MMP = mitochondrial membrane potential, MBWW = modified Biggers, Whitten, and Whittingham.

**Table 6:** Summary of noteworthy results on melatonin supplementation (Mel) for semen storage above 5 °C in various species.

Species	Temp (°C)	Extender	Result	Reference
Ram	39	Saline medium	Higher maturation rate at 1 μm than 10 nm and 100 pm at 3 h	[104]
			Higher % of capacitated cells at 10 nm than 1 um and 100 pm at 3 h	
			Higher fertilisation rate at 100 pm than 1 μm and 100 pm at 3 h	
	5	TRIS extender + EY ^1^	Higher PM at 1 mM than at 0.1 and 3 mM at 48 h	[97]
	4	Tris-based extender	Higher plasma membrane integrity at 0.1 mM than at 0.05 mM, 0.2 mM and 0.4 mM at 120 h	[18]
			Higher MDA at 0.4 mM than at 0.1 mM at 120 h	
Boar	17	VL ^1^	Higher TM at 1 μM than at 0 μM at 7 h	[105]
Buffalo	39	TALP ^1^	Higher fertilisation at 500 mM when compared to 250 mM and 1000 μM at 18 h	[19]
			Higher TM at 50 pm when compared to 100 pm, 200 pm and 1 μm at 18 h	

^1^ VL = Vitasem LD Magapor, Zaragoza, Spain, EY = egg yolk, MMP = mitochondria membrane potential, SM = skim milk, TALP = Tyrode’s albumin lactate pyruvate.

## Data Availability

Not applicable.
